# *TGFB1* genetic polymorphisms and coronary heart disease risk: a meta-analysis

**DOI:** 10.1186/1471-2350-13-39

**Published:** 2012-05-18

**Authors:** Yingchang Lu, Jolanda MA Boer, Roza M Barsova, Olga Favorova, Anuj Goel, Michael Müller, Edith JM Feskens

**Affiliations:** 1 Division of Human Nutrition, Wageningen University and Research Center, PO Box 8129, 6700, EV, Wageningen, The Netherlands; 2 National Institute for Public Health and the Environment (RIVM), PO Box 1, 3720, BA, Bilthoven, The Netherlands; 3 Russian Cardiology Scientific and Production Center, Russian State Medical University, 121552, Moscow, Russia; 4 Department of Cardiovascular Medicine, University of Oxford, Oxford, UK

## Abstract

**Background:**

Genetic variations in *TGFB1* gene have been studied in relation to coronary heart disease (CHD) risk, but the results were inconsistent.

**Methods:**

We performed a systematic review of published studies on the potential role of *TGFB1* genetic variation in CHD risk. Articles that reported the association of *TGFB1* genetic variants with CHD as primary outcome were searched via Medline and HuGE Navigator through July 2011. The reference lists from included articles were also reviewed.

**Results:**

Data were available from 4 studies involving 1777 cases and 7172 controls for rs1800468, 7 studies involving 5935 cases and 10677 controls for rs1800469, 7 studies involving 6634 cases and 9620 controls for rs1982073, 5 studies involving 5452 cases and 9999 controls for rs1800471, and 4 studies involving 5143 cases and 4229 controls for rs1800472. The pooled odds ratios (ORs) for CHD among minor T allele carriers of rs1800469, minor C allele carriers of rs1982073, and minor C allele carriers of rs1800471 versus homozygous major allele carriers was 1.14 (95% confidence interval [CI]: 1.05-1.24), 1.18 (95% CI: 1.04-1.35), and 1.16 (95% CI: 1.02-1.32), respectively. No substantial heterogeneity for ORs was detected among the included Caucasian populations for all SNPs. However, for rs1800471, the statistical significance disappeared after adjusting for potential publication bias. No significant association was found between rs1800468 and rs1800472 variants and CHD risk.

**Conclusion:**

Minor allele carriers of two genetic variants (rs1800469 and rs1982073) in *TGFB1* have a 15% increased risk of CHD.

## Background

Transforming growth factor-β1 (TGFβ1) is a ubiquitously expressed multifunctional cytokine that is involved in many physiological and pathological processes. TGFβ1 has been demonstrated to be of fundamental importance in the development, physiology and pathology of the vascular system. Research into the mechanisms of TGFβ1 signaling over the past two decades has led to the development of a well-accepted canonical signaling cascade involving heterotetrameric complexes of type I and type II serine/threonine-kinase transmembrane receptors together with Smad transcription factors that act as intracellular signaling effectors. However, the exact mechanisms by which TGFβ1 signaling exerts its effects within the vasculature are still incompletely understood [1-4]. According to the literature [2-7], TGFβ1 can be secreted by several cell types, including peripheral blood mononuclear cells, macrophages, platelets, endothelial cells, vascular smooth muscle cells (VSMCs), myofibroblasts, and renal cells. Its regulatory function on the vessel wall is directed at endothelial cells, VSMC and extracellular matrix [1-3,5-8]. Although the role of TGFβ1 in the pathogenesis of atherosclerosis is being recognized, the association between plasma TGFβ1 levels and coronary heart disease (CHD) risk is still controversial [6,9-11]. There may be several explanations for the controversy: 1), TGFβ1 is a bimodal regulator of both endothelial cells and VSMC proliferation, depending on local TGFβ1 levels, cell density, and/or membrane TGFβ receptors [1,2,5,7,8,12]; 2), different pathophysiological stages of CHD may differentially affect the biological effects of TGFβ1 [1,3,10]; and 3), circulating TGFβ1 levels may not reflect the real vascular interstitial TGFβ1 levels that are directly involved in the pathogenesis of CHD [3-5,13]. Also, animal-model studies of CHD reported inconsistent findings on the role of TGFβ1 in CHD development. This might, however, be due to the dysregulated systemic immune function from different methods used, i.e. injecting TGFβ1 antibodies, infusing a soluble TGFβ receptor, or using transgenic or knockout mice [6,8].

Although the amino acid sequence of the active form of TGFβ1 is highly conserved across mammalian species [7,14,15], common *TGFB1* genetic variations that could cause variable constitutive or induced expression of *TGFB1* or protein structural changes and, as a result, changed TGFβ1 activity, have been identified. They include rs1800468 (−800 G/A) and rs1800469 (−509 C/T) in the promoter region, rs1982073 (868 T/C, Leu10Pro) and rs1800471 (913 G/C, Arg25Pro) in the signal peptide region, and rs1800472 (11929 C/T, Thr263Ile) in the region encoding the precursor part of the protein [4,15-19]. These genetic variants are generally in strong linkage disequilibrium (LD) with each other, and this DNA LD block covers the whole 5′ proximal region of the *TGFB1* gene in Caucasian populations [4,14,16,17]. The minor alleles of these genetic variants or the haplotypes where the minor alleles are located, were associated with increased CHD risk in some [11,16,20], but not all studies [10,14,21,22], and even an opposite association has been observed [23]. This may partly be explained by a relatively small sample size, different CHD endpoints and/or different study populations in each of the published studies. Demonstrating an association may require a much larger number of subjects, which may be beyond the resource of one single study. Multiple replicated loci have recently been identified from genome-wide association (GWA) studies of CHD. However, they together explain only a small part of its heritability [24,25]. It has been suggested that the adopted highly stringent statistical criteria and/or the imperfect coverage of genetic variants by current GWA studies might prevent the discovery of potential loci associated with CHD risk [26]. No meta-analysis describing *TGFB1* genetic variants in relation to CHD risk exists; therefore, we performed a meta-analysis of published data that tested for *TGFB1* genetic variants associated with CHD risk.

## Methods

### Selection criteria

For inclusion, studies 1) had to be case–control or cohort in design, 2) examined the association between *TGFB1* gene polymorphisms and primary outcomes of CHD, coronary artery disease or myocardial infarction (MI), 3) used validated coronary heart disease phenotypes (diagnostic criteria included angiographical confirmation; elevations of cardiac enzymes, changes of electrocardiographic and clinical symptoms according to the World Health Organizations criteria; a documented history of coronary artery bypass graft, percutaneous transluminal coronary angioplasty, or percutaneous coronary intervention), and 4) involved unrelated participants.

### Search strategy

All studies reporting on the association between *TGFB1* gene polymorphism and CHD risk published before July 2011 were identified by comprehensive electronic searches of Medline and HuGENet. Terms used for the searches were “TGFB1,” “ischemic heart disease,” “coronary heart disease,” “coronary artery disease,” “acute coronary syndrome,” “myocardial infarction,” and “angina pectoris” combined with “gene,” “genetic,” “variant,” “mutation” or “polymorphism.” Hand searches for related articles among the reference lists of included articles were also performed. If essential information of a study was not presented in the publication, authors were contacted for details. The study was excluded if the information could not be obtained. In addition, the relevant data from the two latest large-scale meta-analyses of CHD GWA studies (PROCARDIS [25] and CARDIoGRAM [24]; Additional file ( 1)) were also included for part of the analysis.

### Data extraction

The first author, published year, country, study population, mean age of participants, gender distribution, study design, sample size, outcome, diagnostic criteria, genotyping method, characteristics of the controls, allele frequencies, and genotype distributions were extracted. In PROCARDIS [25] and CARDIoGRAM [24], the studied SNPs were not available in the genome-wide genotyping assay and had to be imputed. The SNPs imputed with high quality (MACH_R^2^ > 0.3) were included in the analyses.

### Statistical analysis

Deviance from Hardy-Weinberg equilibrium (HWE) was assessed for the controls of each study using Fisher’s exact test. For an Iranian study [11], only data on rs1982073 was utilized in the meta-analysis because other SNPs deviated from HWE. Genotype distributions of controls for studies with case–control design or the entire group for studies with cohort design were used to estimate the frequency of the putative risk allele for each SNP using the inverse variance method (Additional file 2) [27]. Crude ORs with 95% confidence intervals (CI) were used to evaluate the association between genetic polymorphisms and CHD risk. Pooled ORs were calculated for several genetic models, i.e. the co-dominant model, the dominant model, and the recessive model. Since the co-dominant model effects (or additive model effects) cannot be straightforward calculated from the extracted summary data from each study, we presented the effects of two groups of genotype comparison (Additional file 2). Since the using of best-guess genotype from the genotype imputation process can lead to both false positives and loss of power [28], the allele effects from the PROCARDIS [25] and CARDIoGRAM [24] studies were only included in part of the co-dominant model analyses (Additional file 2). Statistical heterogeneity in the ORs across studies was assessed with the Q-test. If there was heterogeneity, ethnicity as source of heterogeneity was explored by pooling the data from Caucasian populations only. If there was no heterogeneity, the fixed-effect model was used to evaluate the overall gene effect; otherwise, the random-effect model was used. Presence of publication bias was explored with Begg’s funnel plot and Egger’s regression test. If potential publication bias existed, the Duval and Tweedie nonparametric “trim and fill” method [29] was used to adjust for it. All reported p values were two-tailed, and statistical significance was defined at the α = 0.05 level. All analyses were performed with the R metafor package [30].

## Results

### Study inclusion and characteristics

Fifteen citations were identified through the original literature search; none were meta-analyses [10,11,14,16,20-23,31-37]. After full review, two studies were excluded because they were conducted in patient cohorts with a composite end-point that included CHD [31,32]. Three studies (two European [10,33] and one Japanese [34]) were conducted without “proper” controls of no CHD history and were also excluded. One additional Chinese study met the inclusion criteria but was excluded due to unavailability of essential information even after contacting the authors [35]. The 9 remaining studies, together with PROCARDIS [25] and CARDIoGRAM [24] studies, were included in the meta-analysis (Table 1) [11,14,16,20-23,36,37].

**Table 1:** **Characteristics of studies included in the meta-analysis**^a^

**Study**	**Country**	**Design**	**Cases**				**Controls**					**Outcome**
			**Genotypes**					**Genotypes**			***p*_HWE**^**c**^	
**rs1800468 (−800 G/A)**			*N*	GG	AG	AA	*N*	GG	AG	AA		
Crobu et al, 2008 [22]	Italy	CC	201	175	25	1	201	168	31	2	0.65	MI
Sie et al, 2006 [21]	Netherlands	CO	358	288	66	4	6098	5071	984	43	0.58	MI
Syrris et al,1998 [14]	England	CC	655	541	110	4	244	207	36	1	1	CAD
Cambien et al,1996 [16]	FR and NIE	CC	563	472	88	3	629	534	89	6	0.28	MI
**rs1800469 (−509 C/T)**				CC	CT	TT		CC	CT	TT		
Sudomoina et al, 2010 [36]^b^	Russia	CC	264	77	150	37	212	90	103	19	0.22	MI
Drenos et al, 2009 [37]	England	CC	240	120	100	20	2143	1090	885	168	0.56	CAD
Crobu et al, 2008 [22]	Italy	CC	201	67	87	47	201	80	92	29	0.76	MI
Koch et al, 2006 [20]	Germany	CC	3657	1581	1659	417	1211	564	508	139	0.13	MI
Sie et al, 2006 [21]	Netherlands	CO	355	171	156	28	6037	3043	2441	553	0.05	MI
Syrris et al, 1998 [14]	England	CC	655	301	284	70	244	124	97	23	0.54	CAD
Cambien et al, 1996 [16]	FR and NIE	CC	563	240	257	66	629	263	297	69	0.29	MI
**rs1982073 (868 T/C)**				TT	TC	CC		TT	TC	CC		
Najar et al, 2011 [11]	Iran	CC	900	301	424	175	900	395	403	102	1	MI
Crobu et al, 2008 [22]	Italy	CC	201	55	88	58	201	69	101	31	0.66	MI
Koch et al, 2006 [20]	Germany	CC	3657	1235	1802	620	1211	458	565	188	0.55	MI
Sie et al, 2006 [21]	Netherlands	CO	343	135	164	44	5844	2322	2698	824	0.37	MI
Yokota et al, 2000 [23]	Japan	CC	315	89	185	41	591	149	295	147	1	MI
Syrris et al, 1998 [14]	England	CC	655	242	306	107	244	102	109	33	0.68	CAD
Cambien et al, 1996 [16]	FR and NIE	CC	563	181	277	105	629	225	297	107	0.62	MI
**rs1800471 (913 G/C)**				GG	GC	CC		GG	GC	CC		
Drenos et al, 2009 [37]	England	CC	234	187	45	2	2071	1723	331	17	0.78	CAD
Koch et al, 2006 [20]	Germany	CC	3657	3149	486	22	1211	1063	141	7	0.33	MI
Sie et al, 2006 [21]	Netherlands	CO	343	297	45	1	5844	4992	823	29	0.51	MI
Syrris et al, 1998 [14]	England	CC	655	558	95	2	244	214	30	0	0.61	CAD
Cambien et al, 1996 [16]	FR and NIE	CC	563	464	92	7	629	546	81	2	1	MI
**rs1800472 (11929 C/T)**				CC	CT	TT		CC	CT	TT		
Drenos et al, 2009 [37]	England	CC	241	234	7	0	2145	2052	89	4	0.02	CAD
Koch et al, 2006 [20]	Germany	CC	3657	3421	231	5	1211	1138	72	1	1	MI
Syrris et al, 1998 [14]	England	CC	655	622	33	0	244	237	7	0	1	CAD
Cambien et al, 1996 [16]	FR and NIE	CC	590	563	27	0	629	585	42	2	0.20	MI

FR and NIE, France and Northern Ireland; CC, case–control; CO, cohort; MI, myocardial infarction; CAD, coronary artery disease.

a, For rs1800468, rs1982073 and rs1800471, 10090 additional subjects were included for the co-dominant model analysis from the PROCARDIS study [25]; for rs1800469, additional 10090 and 80016 subjects were included for the co-dominant model analysis, respectively from the PROCARDIS study [25] and the CARDIoGRAM study [24] (Additional file 2).

b, Additional unpublished data have been included.

c, The *p* values for Hardy-Weinberg equilibrium were derived from Fisher’s exact test.

### Quantitative synthesis

None of the genetic variants were associated with CHD risk when applying a recessive model (data not shown). Rs1800468 and rs1800472 were not associated with CHD in either co-dominant or dominant model (Additional file 2).

**Figure 1:**
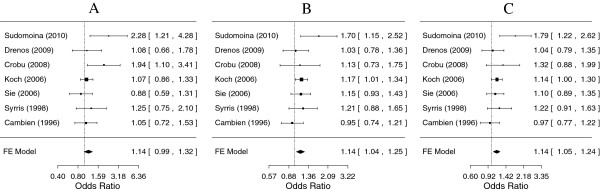
**Meta-analysis for coronary heart disease risk depending on the rs1800469 (−509 C/T) polymorphism in the *TGFB1 *gene.** ORs and corresponding 95% confidence intervals (CIs) are shown. Fixed effects were reported because no significant heterogeneity between studies was observed. **A**. Comparison of the homozygous TT genotype with the wild type CC genotype (*p* = 0.08); **B**. Comparison of the heterozygous CT genotype with the wild type CC genotype (*p* = 0.004); **C**. Comparison of the TT + CT genotype with the wild type CC genotype (*p* = 0.003).

**Figure 2:**
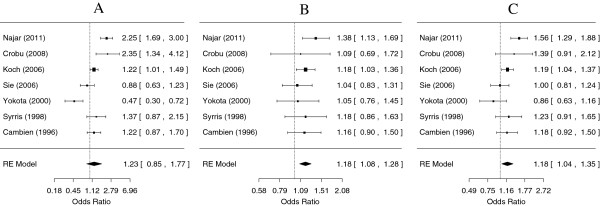
**Meta-analysis for coronary heart disease risk depending on the rs1982073 (868 T/C) polymorphism in the *TGFB1 *gene.** ORs and corresponding 95% confidence intervals (CIs) are shown. **A**. Comparison of the homozygous CC genotype with the wild type TT genotype (random-effect model, *p* = 0.26); **B**. Comparison of the heterozygous TC genotype with the wild type TT genotype (random-effect model, *p* = 0.0002); **C**. Comparison of the CC + TC genotype with the wild type TT genotype (random-effect model, *p* = 0.01).

For rs1800469, both the CT genotype in the co-dominant model and the presence of the minor T allele in the dominant model conferred a risk for CHD when compared to the common CC genotype (OR = 1.14, 95% CI: 1.04-1.25; and OR = 1.14, 95% CI: 1.05-1.24, respectively). The TT genotype conferred a non-significant risk of similar magnitude (Figure 1). For rs1982073, the TC genotype conferred a risk for CHD in the co-dominant model (OR = 1.18, 95% CI: 1.08-1.28), but the CC genotype did not when compared to the common TT genotype (Figure 2). Under a dominant model, the presence of the minor C allele was associated with a 1.18 times increased risk for CHD (Additional file 2).

**Figure 3:**
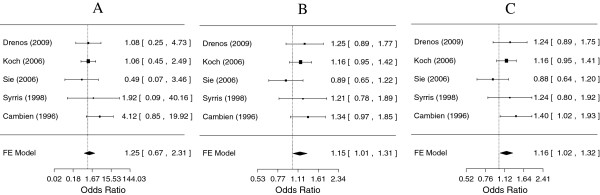
**Meta-analysis for coronary heart disease risk depending on the rs1800471 (913 G/C) polymorphism in the *TGFB1 *gene.** ORs and corresponding 95% confidence intervals (CIs) are shown. Fixed effects were reported because no significant heterogeneity between studies was observed. **A**. Comparison of the homozygous CC genotype with the wild type GG genotype (*p* = 0.49); **B**. Comparison of the heterozygous GC genotype with the wild type GG genotype (*p* = 0.03); **C**. Comparison of the GC + CC genotype with the wild type GG genotype (*p* = 0.02).

For rs1800471, both the GC genotype in the co-dominant model and the presence of the minor C allele in the dominant model conferred a risk for CHD when compared to the common GG genotype (OR = 1.15, 95% CI: 1.01-1.31; and OR = 1.16, 95% CI: 1.02-1.32, respectively). The CC genotype conferred a 1.25 times increased risk, but this was not statistically significant (Figure 3).

After adjusting for multiple testing using Bonferroni correction, all significant associations for rs1800469 and rs1982073 under the co-dominant and dominant models remained. However, for rs1800471, associations were no longer statistically significant (*p* > 0.017 in Additional file 2). When the relevant allele effects from the PROCARDIS [25] and CARDIoGRAM [24] studies were included in the co-dominant model analyses, the aforementioned associations attenuated; however, the association for rs1982073 persisted (Additional file 2).

No substantial heterogeneity for the ORs was detected among the included Caucasian populations (Additional file 2). For rs1982073, however, some heterogeneity existed between Caucasian populations and non-Caucasian populations with regard to both CC and CC + TC vs. TT contrasts (Additional file 2).

### Sensitivity analysis and publication bias

To evaluate the influence of the individual studies on the pooled ORs for rs1800469, rs1982073 and rs1800471, each time, a single study involved in the meta-analysis was deleted. The Rotterdam study [21] and the Japanese study [23] tended to attenuate the pooled ORs in the co-dominant model for rs1800469 and rs1982073, respectively (Additional file 3). No substantial alteration in the observed pooled ORs was observed for rs1800471 (Additional file 3). Begg’s funnel plot and Egger’s regression test were performed to assess potential publication bias for rs1800469, rs1982073 and rs1800471. Although the *P* values for Egger’s regression tests for all investigated models were > 0.05, Begg’s funnel plot still suggested a certain degree of publication bias, potentially from small studies with significant positive results (Additional file 4). After performing the “trim and fill method” to adjust for potential publication bias, the results for rs1800469 and rs1982073 did not change significantly (data not shown). However, for rs1800471, the statistical significance for both co-dominant and dominant models disappeared (*P* = 0.07).

## Discussion

Several studies have been carried out to test the hypothesis that genetic polymorphisms in the *TGFB1* gene including rs1800468, rs1800469, rs1982073, rs1800471 and rs1800472 might be associated with CHD risk, but data have yielded conflicting results. Possible concerns in genetic association studies are that a positive association might be spurious, while a negative result might be due to a small sample size. In this meta-analysis, we incorporated all eligible studies to date and provided some evidence that rs1800469 and rs1982073 in the *TGFB1* gene are associated with CHD risk in Caucasian populations. The inconsistency between the previously reported results for these SNPs might be due to the small sample sizes in most of the studies, especially in combination with a modest effect.

Circulating TGFβ1 levels are predominantly under genetic control with a heritability of 0.54 [17]. Both the CHD-associated minor risk alleles of rs1800469 and rs1982073 correlate with an increase in gene expression, TGFβ1 secretion, and plasma TGFβ1 levels [4,11,15,17,19,23]. These similar observations might be due to the strong LD between them [4,14,16,17]. Shah et al. [18] demonstrated exclusively *in vivo* and *in vitro* recruitment of transcription regulator AP1 to -509 C (the major non-risk allele of rs1800469) leading to transcriptional repression of the *TGFB1* gene. However, the exact functional variant in this gene region merits further identification. In support of the aforementioned positive association between CHD risk alleles of *TGFB1* and increased TGFβ1 production, increased TGFβ1 levels were observed in different stages of plaque development in some histological studies [7,38-40]. In addition, enhanced TGFβ1 signalling is established to cause cartilaginous metaplasia of vascular media and progressive intima-media thickening after vascular injuries [2-5,7,8,12]. Interestingly, an increased TGFβ1 regulated gene expression was observed in both atherosclerotic and restenotic lesions [41]. Recently, the TGFβ1 signalling pathway is suggested to be involved in the genetic determining of CHD for the most replicated 9p21.3 locus [42-44]. A genetic variant in the *SMAD3* gene that encodes one of the downstream activating transcriptional mediators (Smad3) of TGFβ1 signalling [1,2] was associated with CHD risk in a GWA study [44], which was recently replicated in a large-scale meta-analysis of CHD studies [45].

It has previously been shown that abnormal enlargement of human coronary arteries (positive remodelling and aneurysmal coronary lesions) occurs in response to the development of intimal plaque [46-49], which is correlated with future acute coronary syndromes and cardiac events [50,51]. A strong heritable component (*h*^*2*^ = 0.52) of such abnormal enlargement of the coronary artery in the pathogenesis of coronary artery disease was observed, especially in the proximal coronary artery [52,53]. Interestingly, the 9p21.3 locus is also associated with increased risk of abdominal aortic aneurysm [54-56] and intracranial aneurysm [57,58]. Recently, high plasma TGFβ1 levels have been implicated in the manifestation of aortic root dilation in Marfan syndrome [6,13,59]. Furthermore, genetic variations along the TGFβ1 signalling pathway are associated with coronary artery aneurysm formation and aortic root dilation in Kawasaki diseases [60], whereas mutations in genes of TGFβ1 signalling pathway (*TGFBR1*, *TGFBR2*[61,62], and *SMAD3*[63]) or TGFβ1 inhibitor genes [64] are implicated in familial or syndromic forms of thoracic aortic aneurysms and dissection. Taken together, this points at altered vascular remodelling from increased TGFβ1 signalling in the pathogenesis of CHD. However, given the fact that TGFβ1 is produced by multiple lineages of resident cells in vascular wall and atherosclerotic lesion and the fact that it acts in an autocrine, paracrine, and endocrine fashion [4], it has been very difficult to pinpoint the exact cellular sources of TGFβ1 that are relevant for the pathogenesis of CHD. More research on this topic is warranted.

Some limitations of this meta-analysis should be acknowledged. First, a relatively small number of studies for each SNP was included, and therefore we cannot rule out heterogeneity completely in Caucasian populations although most of the *P* values for Q-tests were > 0.05. Second, the results in the co-dominant model for rs1800469 and rs1982073 were dominated by the Rotterdam [21] and the Japanese study [23] as shown by the sensitivity analysis. However, this did not affect our main conclusions. Third, the potential publication bias of relatively small sample-sized studies might have affected the results, as there might be eligible studies with negative results that were not published. In the context of these limitations, our current results should be interpreted with caution.

## Conclusion

In conclusion, the present study demonstrates an association between rs1800469 and rs1982073 in the *TGFB1* gene and CHD risk in Caucasian populations. Enhanced TGFβ1 signalling may therefore be involved in the pathogenesis of CHD.

## Abbreviations

CHD: Coronary heart disease; CI: Confidence interval; GWA: Genome-wide association; HWE: Hardy-Weinberg equilibrium; LD: Linkage disequilibrium; ORs: Odds ratios; VSMC: Vascular smooth muscle cells.

## Competing interests

The authors declare that they have no competing interests.

## Authors’ contributions

YL, JMB, RMB, OF, AG, CARDIoGRAM Consortium, MM, and EJF have made substantial contributions to conception and design, or acquisition of the data, or analysis and interpretation of data; YL, JMB, RMB, OF, and EJF have been involved in drafting the manuscript or revising it critically for important intellectual content; and all authors have given final approval of the version to be published. All authors read and approved the final manuscript.

Members and affiliations of the CARDIoGRAM Consortium are listed in the Additional files.

## Pre-publication history

The pre-publication history for this paper can be accessed here:

http://www.biomedcentral.com/1471-2350/13/39/prepub

## Supplementary Material

Additional file 1 Additional acknowledgments for the PROCARDIS study and the CARDIoGRAM study. Click here for file

Additional file 2 **Table S1.** Summary of the meta-analysis of studies examining the association between TGFB1 polymorphisms and coronary heart disease risk. Click here for file

Additional file 3 **Table S2.** Results from the leave-1-out sensitivity analysis. Click here for file

Additional file 4 **Figure S1.** Funnel plots with pseudo 95% confidence intervals for rs1800469 analysed according to different genotype contrasts. A. Comparison of the homozygous TT genotype with the wild type CC genotype (fixed-effect model, *p* for Egger’s regression test = 0.05); B. Comparison of the heterozygous CT genotype with the wild type CC genotype (fixed-effect model, *p* for Egger’s regression test = 0.61); C. Comparison of the TT+CT genotype with the wild type CC genotype (fixed-effect model, *p* for Egger’s regression test = 0.22). **Figure S2.** Funnel plots with pseudo 95% confidence intervals for rs1982073 analysed according to different genotype contrasts. A. Comparison of the homozygous CC genotype with the wild type TT genotype (random-effect model, *p* for Egger’s regression test = 0.94); B. Comparison of the heterozygous TC genotype with the wild type TT genotype (random-effect model, *p* for Egger’s regression test = 0.50); C. Comparison of the variant genotype of CC+TC with the wild type TT genotype (random-effect model, *p* for Egger’s regression test = 0.71). **Figure S3.** Funnel plots with pseudo 95% confidence intervals for rs1800471 analysed according to different genotype contrasts. A. Comparison of the homozygous CC genotype with the wild type GG genotype (fixed-effect model, *p* for Egger’s regression test = 0.75); B. Comparison of the heterozygous GC genotype with the wild type GG genotype (fixed-effect model, *p* for Egger’s regression test = 0.89); C. Comparison of the variant genotype of CC+GC with the wild type GG genotype (fixed-effect model, *p* for Egger’s regression test = 0.83). Click here for file
