# Supplementary material for: TGFB1 genetic polymorphisms and coronary heart disease risk: a meta-analysis
Source: BMC Med Genet. 2012 May 18;13:39. doi: 10.1186/1471-2350-13-39 (PMC3497590; doi:10.1186/1471-2350-13-39)
Supplement: Additional file 1 — Additional acknowledgments for the PROCARDIS study and the CARDIoGRAM study. [file 1471-2350-13-39-S1.doc]

**Additional file 1**

Table 1 Summary of the meta-analysis of studies examining the association between *TGFB1* polymorphisms and coronary heart disease risk.

| SNP | MA(MAF) | OR (95% CI) ^a^ | | | | | | | | |
| --- | --- | --- | --- | --- | --- | --- | --- | --- | --- | --- |
| Rs1800468 | A (8.7%) | AA vs. GG | *p1* | *p2* | AG vs. GG | *p1* | *p2* | (AA+AG) vs GG | *p1* | *p2* |
|  |  | 1.08 (0.52-2.22) | 0.84 | 0.58 | 1.13 (0.95-1.35) | 0.17 | 0.56 | 1.12 (0.95-1.33) | 0.18 | 0.47 |
|  |  |  |  |  | 1.03 (0.93-1.14) ^b^ | 0.52 | 0.47 |  |  |  |
| Rs1800469 | T (31.3%) | TT vs. CC |  |  | CT vs. CC |  |  | (TT+CT) vs CC |  |  |
|  |  | 1.14 (0.99-1.32) | 0.08 | 0.11 | 1.14 (1.04-1.25) | 0.0041 | 0.32 | 1.14 (1.05-1.24) | 0.0029 | 0.19 |
|  |  |  |  |  | 1.07 (1.01-1.13) ^b^ | 0.016 | 0.18 |  |  |  |
|  |  |  |  |  | 1.06 (0.99-1.13) ^c^ | 0.12 | 0.03 |  |  |  |
| Rs1982073 | C (39.4%) ^d^ | CC vs TT |  |  | TC vs. TT |  |  | (CC+TC) vs TT |  |  |
|  |  | 1.23 (0.85-1.77) ^e^ | 0.26 | < 0.0001 | 1.18 (1.08-1.28) ^e^ | 0.0002 | 0.66 | 1.18 (1.04-1.35) ^e^ | 0.012 | 0.02 |
|  |  | 1.22 (1.06-1.40) ^f^ | 0.0057 | 0.06 | 1.15 (1.04-1.27) ^f^ | 0.0084 | 0.91 | 1.16 (1.06-1.28) ^f^ | 0.0021 | 0.58 |
|  |  |  |  |  | 1.07 (1.02-1.13) ^b^ | 0.0107 | 0.08 |  |  |  |
| Rs1800471 | C (7.4%) | CC vs. GG |  |  | GC vs. GG |  |  | (CC+GC) vs. GG |  |  |
|  |  | 1.25 (0.67-2.31) | 0.49 | 0.50 | 1.15 (1.01-1.31) | 0.0335 | 0.45 | 1.16 (1.02-1.32) | 0.0238 | 0.33 |
|  |  |  |  |  | 1.04 (0.96-1.14) ^b^ | 0.3605 | 0.17 |  |  |  |
| Rs1800472 | C (2.4%) | TT vs. CC |  |  | CT vs. CC |  |  | (TT+CT) vs. CC |  |  |
|  |  | 0.58 (0.13-2.63) | 0.48 | 0.68 | 0.98 (0.79-1.21) | 0.83 | 0.14 | 0.97 (0.78-1.20) | 0.76 | 0.09 |

SNP, single nucleotide polymorphism; MA (MAF), minor allele (pooled minor allele frequency); OR (95% CI), pooled odds ratios and corresponding 95% confidence intervals.

a, *p1*, *p*_value of the meta-analysis; *p2*, *p*_value of the Q-test for heterogeneity.

b, Pooled fixed-effects in Caucasian studies (including the PROCARDIS study).

c, Pooled random-effects in Caucasian studies (including the PROCARDIS and CARDIoGRAM studies).

d, Frequency of minor C allele was 37.7% (pooled) in 5 Caucasian studies, 33.7% in the Iranian study, and 49.8% in the Japanese study.

e, Pooled random-effects in all 7 studies.

f, Pooled fixed-effects in 5 Caucasian studies.
