# Supplementary material for: TGFB1 genetic polymorphisms and coronary heart disease risk: a meta-analysis
Source: BMC Med Genet. 2012 May 18;13:39. doi: 10.1186/1471-2350-13-39 (PMC3497590; doi:10.1186/1471-2350-13-39)
Supplement: Additional file 2 — Table S1. Summary of the meta-analysis of studies examining the association between TGFB1 polymorphisms and coronary heart disease risk. [file 1471-2350-13-39-S2.doc]

**Additional file 2**

Table 1 Results from the leave-1-out sensitivity analysis

| SNP | Leave-1-out | OR (95% CI) and p_value of the Q-test for heterogeneity | | | | | |
| --- | --- | --- | --- | --- | --- | --- | --- |
| Rs1800469 |  | TT vs. CC | *p* | CT vs. CC | *p* | (TT+CT) vs CC | *p* |
|  | Rotterdam study (1) | 1.18 (1.01-1.38) | 0.13 | - | - | - | - |
|  | German study (2) | - | - | 1.12 (1.00-1.26) ^b^ | 0.24 | - | - |
| Rs1982073 ^a^ |  | CC vs TT |  | TC vs. TT |  | (CC+TC) vs TT |  |
|  | Japanese study (3) | 1.42 (1.08-1.86) | 0.0003 | - | - | - | - |
|  | German study (2) | - | - | - | - | 1.18 (1.00-1.39) ^b^ | 0.01 |
| Rs1800471 |  | CC vs. GG |  | GC vs. GG |  | (CC+GC) vs. GG |  |
|  | England study (2009) (4) | - | - | 1.14 (0.99-1.31) | 0.33 | 1.15 (1.00-1.32) ^b^ | 0.22 |
|  | German study (2) | - | - | 1.14 (0.96-1.36) | 0.30 | 1.16 (0.99-1.38) | 0.20 |
|  | England study (1998) (5) | - | - | 1.15 (1.00-1.32) ^b^ | 0.31 | - | - |
|  | French study (1996) (6) | - | - | 1.12 (0.97-1.29) | 0.44 | - | - |

SNP, single nucleotide polymorphism; OR (95% CI), pooled odds ratios and corresponding 95% confidence intervals.

a, All 7 studies analysed with a random-effect model.

b, Borderline significance.

References:

(1) Sie MP, Uitterlinden AG, Bos MJ, Arp PP, Breteler MM, Koudstaal PJ, Pols HA, Hofman A, van Duijn CM, Witteman JC. TGF-beta 1 polymorphisms and risk of myocardial infarction and stroke: the Rotterdam Study. *Stroke; a journal of cerebral circulation* 2006;37:2667-2671.

(2) Koch W, Hoppmann P, Mueller JC, Schomig A, Kastrati A. Association of transforming growth factor-beta1 gene polymorphisms with myocardial infarction in patients with angiographically proven coronary heart disease. *Arterioscler Thromb Vasc Biol* 2006;26:1114-1119.

(3) Yokota M, Ichihara S, Lin TL, Nakashima N, Yamada Y. Association of a T29-->C polymorphism of the transforming growth factor-beta1 gene with genetic susceptibility to myocardial infarction in Japanese. *Circulation* 2000;101:2783-2787.

(4) Drenos F, Talmud PJ, Casas JP, Smeeth L, Palmen J, Humphries SE, Hingorani AD. Integrated associations of genotypes with multiple blood biomarkers linked to coronary heart disease risk. *Human molecular genetics* 2009;18:2305-2316.

(5) Syrris P, Carter ND, Metcalfe JC, Kemp PR, Grainger DJ, Kaski JC, Crossman DC, Francis SE, Gunn J, Jeffery S, Heathcote K. Transforming growth factor-beta1 gene polymorphisms and coronary artery disease. *Clin Sci (Lond)* 1998;95:659-667.

(6) Cambien F, Ricard S, Troesch A, Mallet C, Generenaz L, Evans A, Arveiler D, Luc G, Ruidavets JB, Poirier O. Polymorphisms of the transforming growth factor-beta 1 gene in relation to myocardial infarction and blood pressure. The Etude Cas-Temoin de l'Infarctus du Myocarde (ECTIM) Study. *Hypertension* 1996;28:881-887.
