# Supplementary material for: TGFB1 genetic polymorphisms and coronary heart disease risk: a meta-analysis
Source: BMC Med Genet. 2012 May 18;13:39. doi: 10.1186/1471-2350-13-39 (PMC3497590; doi:10.1186/1471-2350-13-39)

Additional file 3

Figure 1 Funnel plots with pseudo 95% confidence intervals for rs1800469 analysed according to different genotype contrasts. A. Comparison of the homozygous TT genotype with the wild type CC genotype (fixed-effect model, *p* for Egger's regression test = 0.05); B. Comparison of the heterozygous CT genotype with the wild type CC genotype (fixed-effect model, *p* for Egger's regression test = 0.61); C. Comparison of the TT+CT genotype with the wild type CC genotype (fixed-effect model, *p* for Egger's regression test = 0.22).

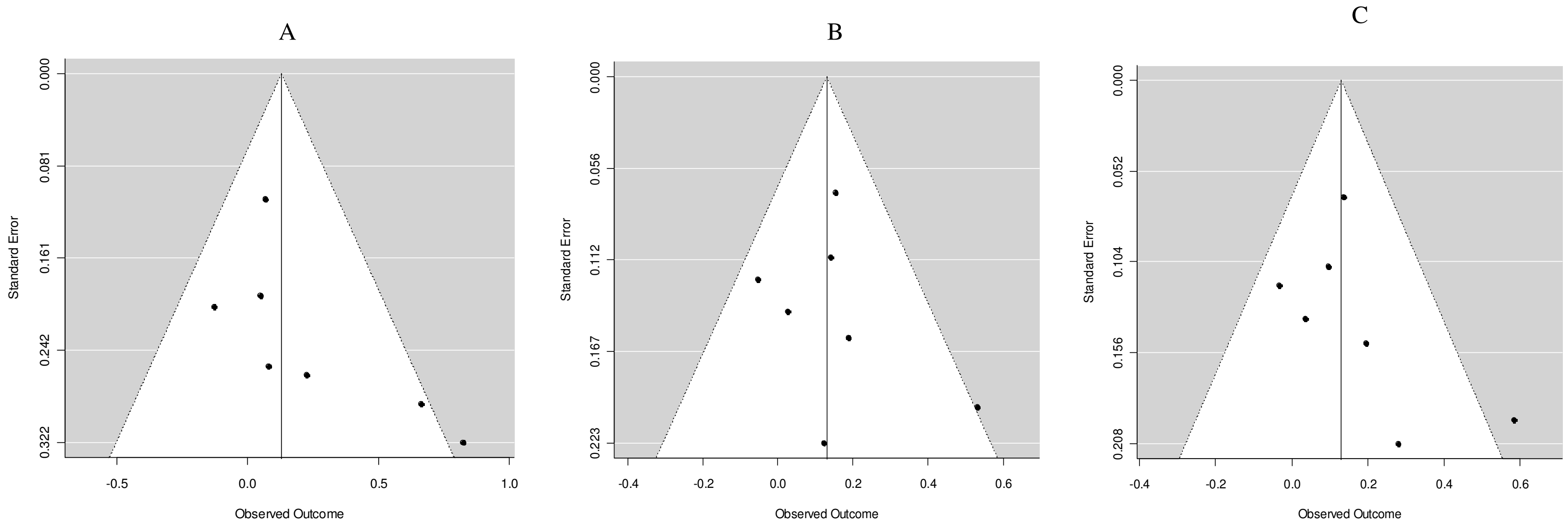

Figure 2 Funnel plots with pseudo 95% confidence intervals for rs1982073 analysed according to different genotype contrasts. A. Comparison of the homozygous CC genotype with the wild type TT genotype (random-effect model, *p* for Egger's regression test = 0.94); B. Comparison of the heterozygous TC genotype with the wild type TT genotype (random-effect model, *p* for Egger's regression test = 0.50); C. Comparison of the variant genotype of CC+TC with the wild type TT genotype (random-effect model, *p* for Egger's regression test = 0.71).

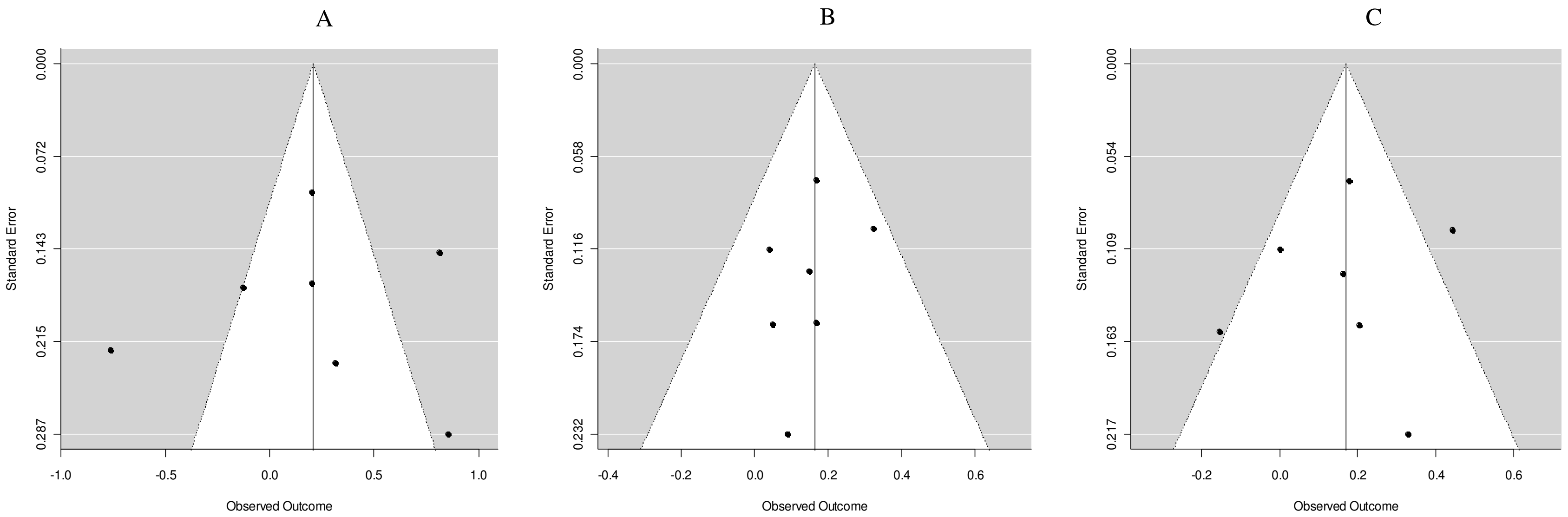

Figure 3 Funnel plots with pseudo 95% confidence intervals for rs1800471 analysed according to different genotype contrasts. A. Comparison of the homozygous CC genotype with the wild type GG genotype (fixed-effect model, *p* for Egger's regression test = 0.75); B. Comparison of the heterozygous GC genotype with the wild type GG genotype (fixed-effect model, *p* for Egger's regression test = 0.89); C. Comparison of the variant genotype of CC+GC with the wild type GG genotype (fixed-effect model, *p* for Egger's regression test = 0.83).

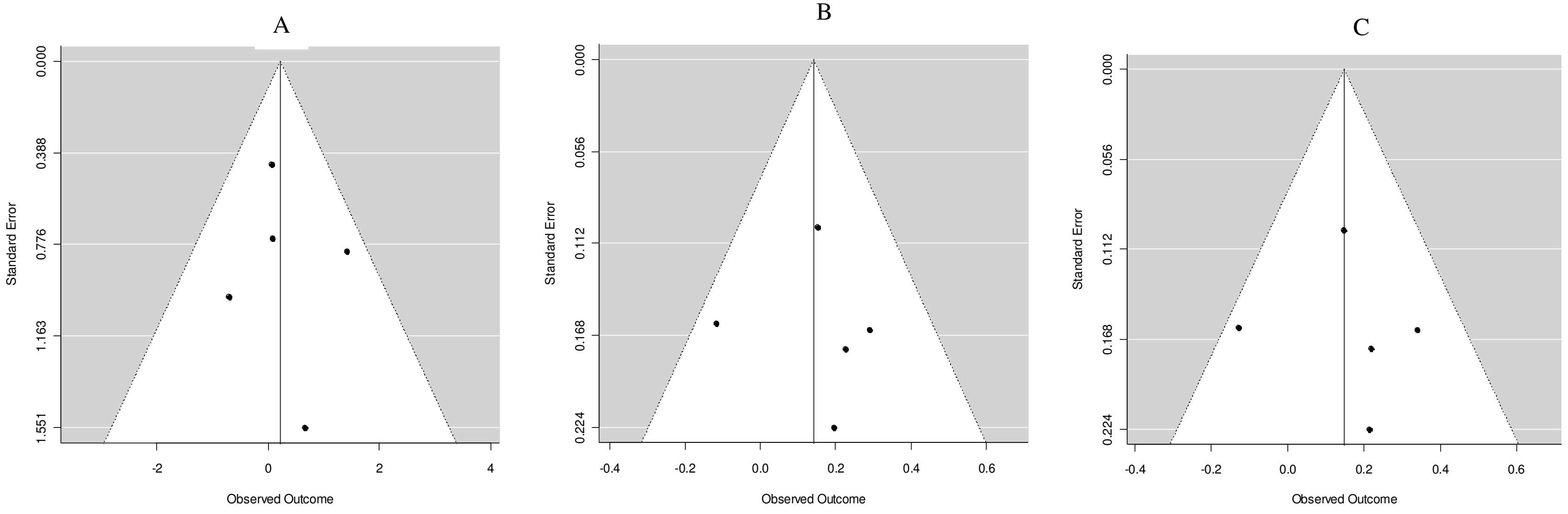

Supplement: Additional file 3 — Table S2. Results from the leave-1-out sensitivity analysis. [file 1471-2350-13-39-S3.pdf]
